# ^90^Sr in teeth of cattle abandoned in evacuation zone: Record of pollution from the Fukushima-Daiichi Nuclear Power Plant accident

**DOI:** 10.1038/srep24077

**Published:** 2016-04-05

**Authors:** Kazuma Koarai, Yasushi Kino, Atsushi Takahashi, Toshihiko Suzuki, Yoshinaka Shimizu, Mirei Chiba, Ken Osaka, Keiichi Sasaki, Tomokazu Fukuda, Emiko Isogai, Hideaki Yamashiro, Toshitaka Oka, Tsutomu Sekine, Manabu Fukumoto, Hisashi Shinoda

**Affiliations:** 1 Department of Chemistry, Tohoku University, Japan; 2 Tohoku University Hospital, Tohoku University, Japan; 3 Graduate School of Dentistry, Tohoku University, Japan; 4 International Research Institute of Disaster Science, Tohoku University, Japan; 5 Graduate School of Agricultural Science, Tohoku University, Japan; 6 Faculty of Agriculture, Niigata University, Japan; 7 Institute for Excellence in Higher Education, Tohoku University, Japan; 8 Institute of Development, Aging and Cancer, Tohoku University, Japan

## Abstract

Here we determined the ^90^Sr concentrations in the teeth of cattle abandoned in the evacuation area of the Fukushima-Daiichi Nuclear Power Plant (FNPP) accident. ^90^Sr activity concentrations in the teeth varied from 6–831 mBq (g Ca)^−1^ and exhibited a positive relationship with the degree of radioactive contamination that the cattle experienced. Even within an individual animal, the specific activity of ^90^Sr (Bq (g Sr)^−1^) varied depending on the development stage of the teeth during the FNPP accident: teeth that were early in development exhibited high ^90^Sr specific activities, while teeth that were late in development exhibited low specific activities. These findings demonstrate that ^90^Sr is incorporated into the teeth during tooth development; thus, tooth ^90^Sr activity concentrations reflect environmental ^90^Sr levels during tooth formation. Assessment of ^90^Sr in teeth could provide useful information about internal exposure to ^90^Sr radiation and allow for the measurement of time-course changes in the degree of environmental ^90^Sr pollution.

The Fukushima-Daiichi Nuclear Power Plant (FNPP) accident released a substantial amount of radioactive nuclides into the atmosphere and caused extensive contamination of the environment^1^,^2^,^3^,^4^,^5^. The radioactivity of the typical fission products was estimated to be 8.2 PBq for ^137^Cs, 9.8 PBq for ^134^Cs, and 0.14 PBq for ^90^Sr^2^,^6^. In June 2011, the Japan Ministry of Education, Culture, Sports, Science, and Technology (MEXT) reported that 0.1−6 kBq m^−2^ of ^90^Sr and 0.3−17 kBq m^−2^ of ^89^Sr were detected in the soil of areas within a 20-km radius from the FNPP (i.e., the former Fukushima evacuation zone)^7^. As ^89^Sr has a relatively short half-life of 50.5 days, its presence suggests that these radionuclides did not originate from global fallout due to nuclear weapons testing, but from the FNPP accident.

The long half-life (28.8 y) and bone-seeking properties of ^90^Sr make it a concerning artificial radionuclide among the fission products found near FNPP. Although radioiodine and caesium are more noticeable in quantity, ^90^Sr can persist in bone with a retention half-life of over 10 years, depending on bone type^8^,^9^,^10^,^11^. Moreover, its daughter nuclide, ^90^Y, emits β-rays (2.28 MeV) that may have adverse effects on the bone marrow. Thus, some attention has been paid to the determination of ^90^Sr content in bone and particularly in teeth. Sr is incorporated into the tooth during calcification. Once incorporated, it remains in enamel and dentine until the tooth falls out or is extracted^12^. Therefore, ^90^Sr activity concentration in a given tooth is a reflection of environmental ^90^Sr contamination levels when the tooth was formed.

Several studies have taken advantage of this phenomenon to understand the long-term effects of nuclear activity on humans. For example, ^90^Sr incorporation into human teeth has been observed after the Techa River region was contaminated by the release of liquid radioactive waste into the river during the early 1950s^8^,^13^,^14^,^15^. Similarly, the deciduous teeth of Swiss children born between 1952 and 2002 exhibited ^90^Sr activity concentrations that correlated with atmospheric rises in ^90^Sr levels, which resulted from nuclear weapons testing during that period^11^. Increases in tooth ^90^Sr activity concentrations following the 1986 Chernobyl accident have also been reported^11^,^16^,^17^. These observations indicate that ^90^Sr activity concentration in teeth is an effective indicator of ^90^Sr contamination levels in the environment. However, while studies have examined ^90^Sr contamination in soil, vegetation, the nearby seawater and fish after the FNPP accident^18^,^19^,^20^,^21^,^22^,^23^,^24^, no studies to date have reported on ^90^Sr activity concentrations in teeth or bones. We thus have little direct data on how much FNPP-related contamination affected animals, which is essential for fully understanding the extent of environmental pollution in the area.

In the aftermath of the FNPP accident, thousands of cattle were abandoned in the evacuation zone. These cattle subsisted on natural food and water in the contaminated environment. Previously, we investigated the activities of ^134^Cs, ^137^Cs, ^110m^Ag, and ^129m^Te in cattle within a 20-km radius around the FNPP, and demonstrated that radioactive Cs concentrations in organs and plasma were dependent on the feeding conditions and the geographic location of the cattle^25^. We also separately examined the effect of radioactive Cs on cattle testes after the FNPP accident^26^. We now expand on these studies by examining ^90^Sr concentrations in cattle teeth and relating them to other measures of environmental pollution.

## Results

### Activity concentration of ^90^Sr in the soil of cattle residence areas

**Figure 1:**
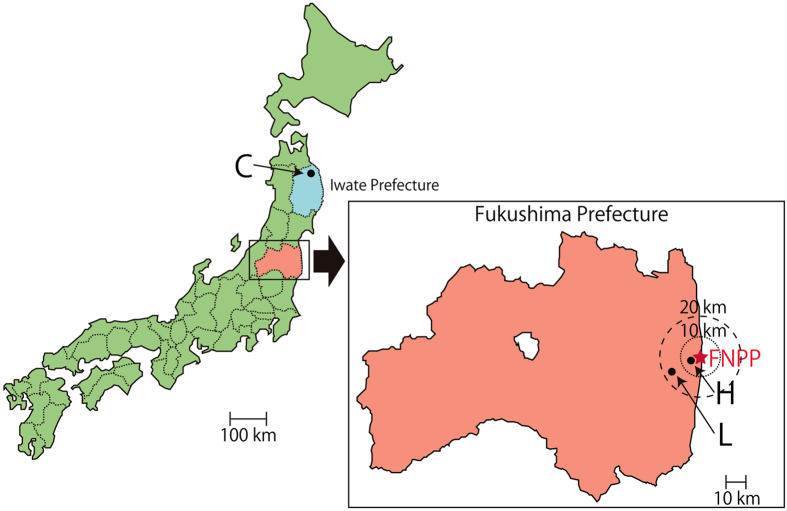
Study sites. FNPP: The Fukushima-Daiichi Nuclear Power Plant. H: High-contamination area (10–30 μSv h^−1^), 5 km west of FNPP. L: Low-contamination area (0.8–1.2 μSv h^−1^), 16 km south-west of FNPP. Areas H and L were in the evacuation zone. C: Control area in Iwate Prefecture, 250 km north of FNPP. The maps were modified from open-access base maps freely available for public and academic use (source: http://maps.gsi.go.jp, from the Geographic Information Authority of Japan).

Figure 1 details the locations of cattle residence after the FNPP accident. Areas H and L are situated in the government-delineated evacuation zone, and their ^90^Sr activity concentrations in soil are 94–1500 Bq m^−2^ (average: 738 Bq m^−2^) and 39–380 Bq m^−2^ (average: 195 Bq m^−2^), respectively^7^. We chose area C in Iwate Prefecture as the control region because it is approximately 250 km north of the nuclear plant and is considered free from FNPP-related ^90^Sr pollution. The activity concentration of ^90^Sr in soil in area C is 95–99 Bq m^−2^ (average: 96 Bq m^−2^)^27^.

### Radioactivity of ^90^Sr in cattle teeth

**Figure 2:**
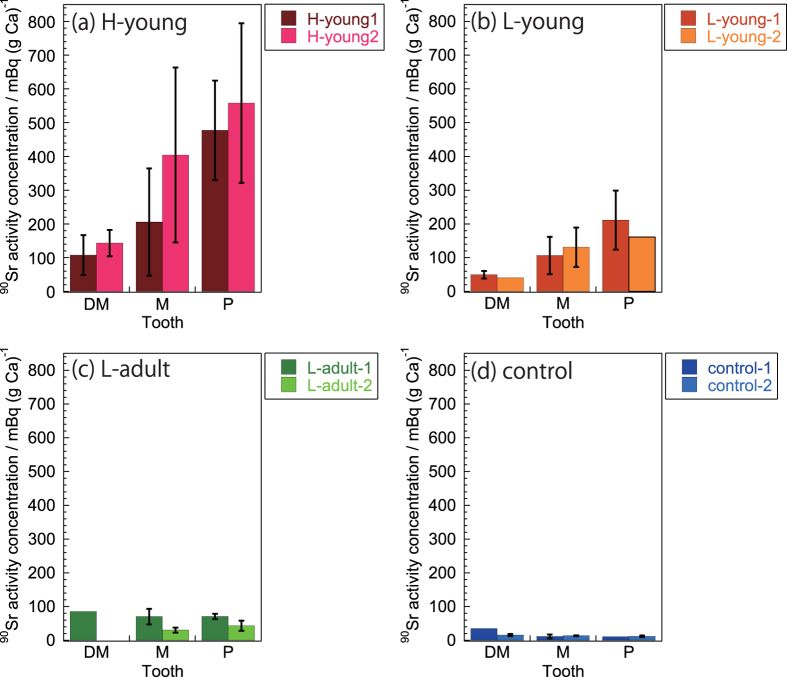
^90^Sr activity concentration in teeth. DM: Deciduous molars (first, second, and third deciduous molar); M: Molars (first, second, and third molar); P: Premolars (first, second, and third premolar). Along the x-axis, the teeth are arranged according to the chronological order of tooth development (DM, M, P). Error bars represent the standard deviation.

**Figure 3:**
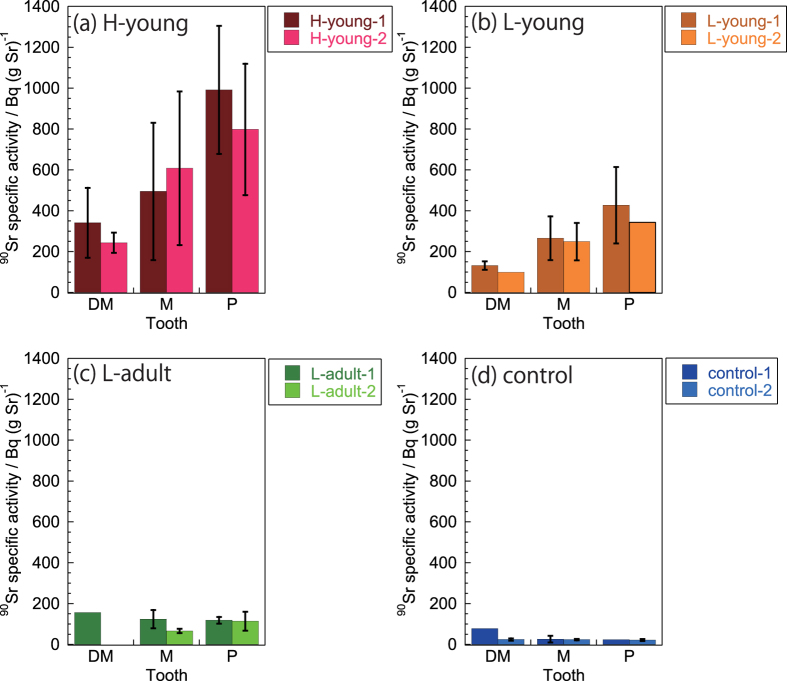
^90^Sr specific activity in teeth. DM: Deciduous molars (first, second, and third deciduous molar); M: Molars (first, second, and third molar); P: Premolars (first, second, and third premolar). Along the x-axis, the teeth are arranged according to the chronological order of tooth development (DM, M, P). Error bars represent the standard deviation.

**Figure 4 f4:**
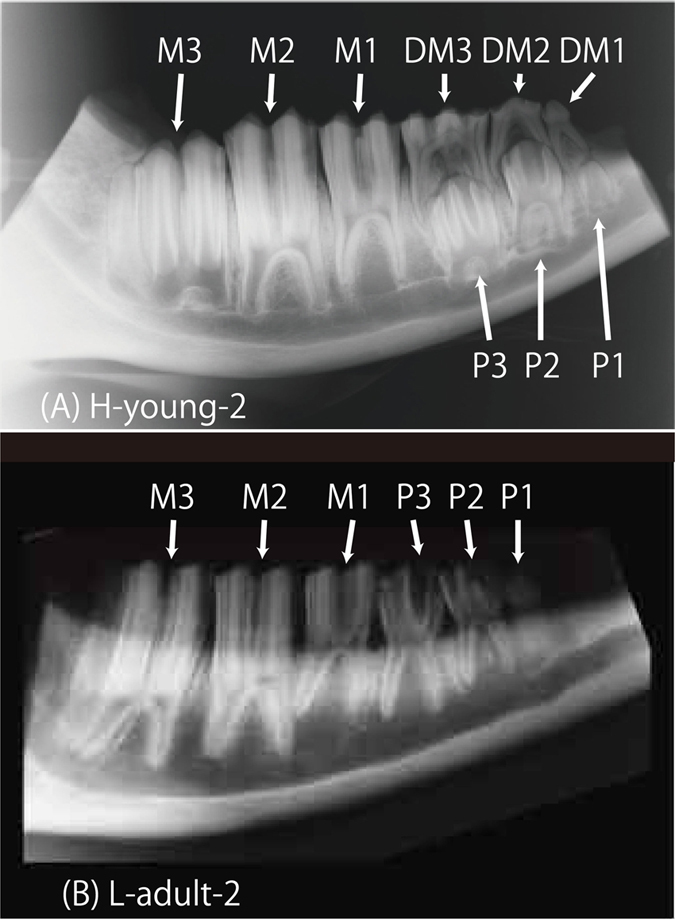
Dental radiographs of mandibular bones from young and adult cattle. (**A**) H-young-2: Deciduous molars were fully developed. Of the molars, the root of the third molar was under active development. The premolars under the deciduous molars were in the early developmental stage. (**A**) L-adult-2: Both molars and premolars were fully developed. Permanent dentition was complete. DM1: First deciduous molar; DM2: Second deciduous molar; DM3: Third deciduous molar; M1: First molar; M2: Second molar; M3: Third molar; P1: First premolar; P2: Second premolar; P3: Third premolar.

1. ^90^Sr activity concentration Figure 2 summarizes the ^90^Sr activity concentrations (^90^Sr activity/amount of Ca) in various teeth (deciduous molars, premolars, and molars (Supplementary Table S1)). We detected ^90^Sr in all examined teeth. Activity concentrations varied significantly with area (p = 0.0000, Kruskal-Wallis test): high ^90^Sr concentrations (61–831 mBq (g Ca)^−1^) were observed in area H, mid-range concentrations (22–311 mBq (g Ca)^−1^ were observed in area L, and the lowest concentrations (6–35 mBq (g Ca)^−1^) were observed in control area C. These ^90^Sr activity concentrations in teeth were significantly correlated with ^90^Sr concentrations in the soil of areas H, L, and C (*ρ* = 0.8441, p < 0.01, Spearman’s rank-order correlation analysis).
2. Specific activity of ^90^Sr Figure 3 shows the specific activities of ^90^Sr (^90^Sr radioactivity/amount of stable Sr) in teeth (Supplementary Table S2). ^90^Sr specific activities were similar to the ^90^Sr activity concentration trends: higher (214–1351 Bq (g Sr)^−1^) and lower (60–641 Bq (g Sr)^−1^) activity were observed for areas H and L, respectively. The lowest specific activity (13–78 Bq (g Sr)^−1^) was observed in teeth from control area C. The specific activity differed significantly across areas H, L, and C (p = 0.0000, Kruskal-Wallis test). Moreover, specific activity was significantly correlated with ^90^Sr concentrations in the soil of areas H, L, and C (*ρ* = 0.8507, p < 0.01, Spearman’s rank-order correlation analysis).
3. Activity concentrations and specific activities of ^90^Sr in teeth at different developmental stages. The four young cattle examined in this study (H-young-1, H-young-2, L-young-1, and L-young-2) were 8 months old when the FNPP accident occurred (Table 1) and their molars ranged across developmental stages: development of the deciduous molars (DM1, DM2, and DM3) were either complete or in the late stage, the molars (M1, M2, and M3) were actively developing, and the premolars (P1, P2, and P3) were still early in development^28^ (Fig. 4a). ^90^Sr activity concentrations and specific activities were low in deciduous molars, higher in molars, and highest in premolars for each individual (p = 0.0006 and 0.0004 for ^90^Sr activity concentration and ^90^Sr specific activity, respectively; Kruskal-Wallis test; Figs 2 and 3). We also determined the activity concentrations and specific activity of ^90^Sr in the teeth of two adult cattle (L-adult-1 and L-adult-2) from area L, that were 22 and 51 months old, respectively, during the FNPP accident. Based on their age, we assumed that development of permanent molars and premolars were complete at that point (Fig. 4b). We found low levels of ^90^Sr activity concentrations (22–91 mBq (g Ca)^−1^) and specific activities (60–166 Bq (g Sr)^−1^) in all adult teeth, but in contrast to young cattle (L-young-1 and L-young-2), no significant differences existed across adult molars and premolars in both ^90^Sr activity concentration and specific activity (p = 0.6310 and 0.3367, respectively; Kruskal-Wallis test).

### Concentration of stable Sr in cattle teeth

We compared stable Sr concentrations in teeth formed before and after the accident (Table 2).

We found that Sr concentrations in the teeth of young cattle in area H and L differed across the deciduous molars, molars, and premolars. The deciduous molars, fully developed before the accident, exhibited low Sr concentrations, while the premolars that developed post-accident exhibited high concentrations. Molars undergoing active development when the accident occurred exhibited mid-range values. The variation in Sr concentrations across teeth was statistically significant in young cattle from areas H and L (p = 0.0211, Kruskal-Wallis test). In contrast, the dentition of adult cattle from area L (molars and premolars; most deciduous molars had fallen out by the time of sampling, see Table 2) was already fully developed when the accident occurred and exhibited no differences in stable Sr concentration (p = 1.0000, Kruskal-Wallis test). We also found no differences among the deciduous molars, molars, and premolars of the young control cattle (p = 0.7488, Kruskal-Wallis test).

## Discussion

The results of our study demonstrated that activity concentrations and specific activities of ^90^Sr in cattle teeth varied in accordance with the degree of ^90^Sr pollution in the cattle residence areas. After the FNPP accident, the cattle were released to the field and subsisted on grasses, leaves, and river or swamp water in the polluted environment. The contamination of natural food and water consumed by the cattle likely contributed to ^90^Sr activity differences we observed in the H-area teeth versus L-area teeth.

Patterns corroborating our results have been reported in cow teeth from 16 contaminated areas in the Mayak region of the former Soviet Union^29^. The study using imaging plates showed that ^90^Sr activity concentrations in the teeth were 0.09–2.96 kBq (g tissue)^−1^ on average and were positively correlated with soil contamination levels (<3.7–185 kBq m^−2^). Although the degree of ^90^Sr contamination in our study areas was much lower than contamination in the Mayak region, we note the similar relationship between environmental ^90^Sr and tooth ^90^Sr activity concentration: ^90^Sr in the teeth faithfully reflects the degree of ^90^Sr pollution in the environment when the tooth was formed.

Small amounts of ^90^Sr were detected in the teeth of control cattle. Similarly, low levels of ^90^Sr have been found elsewhere in Japan even before the accident occurred. For example, ^90^Sr activity concentrations in cattle bones from Hokkaido (located on the northern edge of Japan and relatively far from the FNPP) were approximately 72 mBq (g Ca)^−1^ in 1996^30^ and 26 mBq (g Ca)^−1^ in 2008^31^. Both concentrations are higher than the ^90^Sr activity concentrations in the control teeth (14 ± 7 mBq (g Ca)^−1^) of this study. Possible sources for pre-FNPP radioactivity in Hokkaido are either the Chernobyl accident or nuclear weapons testing. Although ^90^Sr fallout from Chernobyl had been detected in Japan previously, the amount was far less than fallout from nuclear weapons testing^32^. Moreover, increases to ^90^Sr activity concentrations in Hokkaido cattle bones were not observed at the time of the Chernobyl accident^30^. Therefore, the low levels of ^90^Sr activity measured in our controls probably stemmed from the atmospheric nuclear weapons testing conducted during the 1950s–1970s.

In this study, we took advantage of cattle tooth development to examine pre- and post-accident levels of ^90^Sr. Tooth development follows a fixed trajectory that varies across species. In cattle, deciduous molars first form during the prenatal period, followed by molars. Premolars then form during the early postnatal period, with the first premolars (P1) forming in the last stage of dentition, beginning from 12–18 months and completing at 18–24 months^28^. Therefore, cattle younger than 24 months old possess teeth across all dentition developmental stages. Moreover, teeth at early developmental stages during the accident would primarily form under a polluted environment, incorporating large amounts of ^90^Sr. In contrast, the formation of teeth at late developmental stages would be mostly complete during the accident, resulting in the incorporation of less ^90^Sr. Furthermore, ^90^Sr activities in the teeth of adult cattle were low (Fig. 2c) and nearly constant, although the adults had resided in area L, the same location as two of the young cattle.

We observed ^90^Sr activity even in teeth that had fully developed before the accident (i.e., deciduous molars of young cattle in areas H and L, as well as deciduous molars, molars, and premolars of adult cattle in area L). These levels were occasionally higher than levels in control cattle (compare Fig. 2c to Fig. 2d, and Fig. 3c to 3d), although essential incorporation of ^90^Sr was not expected during tooth development. These higher than expected concentrations may have been due to non-specific ^90^Sr adsorption on the tooth surface via contaminated food or water. Alternatively, they could have resulted from the deposition of dental calculus on the tooth surface after the FNPP accident. Furthermore, as described by Tolstykh *et al*.^8^,^14^, ion exchange between the dentine tubule and pulp surfaces with the secondary dentine formation in the pulp could proceed even after complete tooth development. These interactions may also contribute to the higher ^90^Sr activities in adult teeth from area L compared with control teeth.

Data on the stable Sr concentrations in teeth formed pre- and post-accident (Table 2) accorded with the radioactivity data. Specifically, in young cattle of both areas H and L, teeth formed before the accident contained less stable Sr than teeth formed after the accident. The teeth of adult cattle and control cattle, however, did not exhibit a pre- and post-accident difference in stable Sr. Again, similar to our findings for ^90^Sr specific activity, these patterns in the experimental young cattle are likely the result of differences between pre-accident farm feed and the post-accident natural resources that the cattle ingested. Although we did not measure Sr contents in the diets of our subject cattle, our data on stable Sr suggest that the natural grasses, leaves, and water ingested by the cattle likely contain more Sr than their former farm feed. These data suggest that stable Sr concentrations could be also used in conjunction with ^90^Sr specific activity as a metric for environmental Sr levels.

We have shown that ^90^Sr incorporation into teeth is cumulative during tooth development, reflecting the degree of environmental ^90^Sr contamination in that time. Unlike bone, the tooth has essentially no metabolism. Therefore, the ^90^Sr in tooth is a potentially useful indicator for estimating the internal radiation exposure of individuals affected by nuclear activity during their tooth formation periods. Furthermore, by measuring ^90^Sr activities in different teeth, we can take advantage of the various developmental trajectories of animal (including human) dentition and use the known chronologies of individual tooth growth to track time-course changes in the degree of environmental contamination.

## Materials and Methods

### Ethics

This study is one of the national projects associated with the Great East Japan Earthquake that occurred on March 11, 2011. All protocols were approved by the Tohoku University (No.2014KDO037). All methods detailed below were carried out in accordance with these guidelines.

On May 12, 2011, the Japanese Ministry of Agriculture Forestry and Fisheries (MAFF) ordered euthanasia of cattle abandoned in the evacuation zone to prevent radio-contaminated beef from entering consumer products^25^. Euthanasia was carried out by veterinarians belonging to the Livestock Hygiene Service Center (LHSC) of Fukushima Prefecture, in accordance with the Ethical Regulations for Animal Experiments and Related Activities at MAFF. These regulations are based on the June 2007 euthanasia guidelines issued by the American Veterinary Medical Association. The cattle were anesthetized with an intramuscular injection of xylazine hydrochloride (0.2 mg kg^−1^). They were then euthanized via an overdose of intravenous sodium pentobarbital (20 mg kg^−1^), followed by intravenous suxamethonium hydrochloride (2 mg kg^−1^). Before performing euthanasia, veterinarians obtained informed consent from the livestock owners, who were identified from the cattle ear tags. We collected organs and tissues, including mandibular bones, from euthanized cattle with the help of LHSC veterinarians.

### Collection of tooth samples

We selected six cattle (H-young-1, H-young-2, L-young-1, L-young-2, L-adult-1, and L-adult-2) residing in the two FNPP evacuation areas (H and L; Fig. 1) for the current study. Two other cattle (control-1 and control-2) from the uncontaminated area C (Fig. 1) were chosen as controls. The mandibular bones of control cattle, including teeth, were supplied by the Iwate Chikusan Ryutsu Center Co., Ltd. in Iwate Prefecture. The characteristics of the study subjects are summarized in Table 2.

Mandibular bones were dissected from the cattle skulls and radiographs were taken with X-ray equipment (Panoramic Radiograph, Auto-IIIE, Asahi Roentgen Ind. Co., Ltd.) to classify the developmental stages of molar dentition. The deciduous molars, molars, and premolars were then dissected from the mandible and kept in 70% ethanol until needed. We air-dried teeth in a desiccator after removing any surface debris with toothbrushes and dental scalars. The tooth was then crushed with a hammer and powdered using a tissue lyser (TissueLyser II, Qiagen Co., Ltd.). The powdered teeth (1.5 g) were then incinerated in a muffle furnace at 450 °C for 12 h and used for ^90^Sr, stable Sr, and Ca measurements, as described in the following subsection.

In this study, we used the entire tooth for analysis, without separating enamel and dentine. The cattle molar is classified as a hypsodont tooth (*drycodont*), morphologically characterized by extremely high and long crowns that occupy more than 4/5th of the whole tooth^33^. The crown elongates parallel to the growing axis and consists of both enamel and dentine. The latter is metabolically inert, like the enamel, and therefore accumulates Sr in the same way. Additionally, Sr concentration in dentine is slightly higher than in enamel, but the difference is small (70–620 in dentine versus 25–600 μg g^−1^ in enamel^34^). Because both tissues develop almost simultaneously during crown formation, and because the hypsodont tooth primarily consists of the crown, we thought that the relationship between molar development and ^90^Sr accumulation could be properly assessed even without separating enamel and dentine. Further, using the whole tooth allows us to track changes in ^90^Sr deposition over a longer period, because complete tooth formation takes over 2 years, whereas separate parts of the tooth take less time to develop.

### Chemical separation of ^90^Sr in the teeth

Many methods have been developed for the separation of Sr from large amounts of Ca, including co-precipitation, liquid-to-liquid extraction, ion-exchange or extraction chromatography, and combinations of these techniques^24^,^35^,^36^. For this study, we chose the fuming nitric acid method. Although newer methods are less demanding and occasionally result in higher yields, the use of fuming nitric acid remains reliable and robust under conditions of large sample amounts and extremely high quantities of co-existing Ca^35^, which was the case here.

We dissolved 1 g of the incinerated sample in 10 mL of 60% HNO_3_ (analytical grade, Kanto Chemical Co., Inc.), and added 20 mg of Sr^2+^ carrier to the solution. We then added 10 mL of fuming nitric acid (analytical grade, Kanto Chemical Co., Inc.) to precipitate Sr(NO_3_)_2_. We removed the remaining Ca by dissolving the precipitate in 10 mL of distilled water, and again adding 10 mL of fuming nitric acid. The resultant supernatant was discarded. By repeating this procedure two to three times, the Sr within each sample was successfully separated from Ca as Sr (NO_3_)_2_. Sr (NO_3_)_2_ was dissolved in 10 mL of distilled water to form Solution A, which was subjected to further chemical separation from Ra and Pb.

Trace amounts of natural ^226^Ra (half-life: 1600 y), ^228^Ra (half-life: 5.75 y) and ^210^Pb (half-life: 22.3 y) are present in teeth^35^. These radionuclides interfere with accurate β-ray measurement of ^90^Sr. Therefore, these radionuclides were removed via co-precipitation with BaCrO_4_ using the following procedure.

First, 2 mL of acetate buffer solution was added to Solution A. Then, 10 mg of Ba^2+^ was added, followed by dilution with distilled water to a volume of 20 mL. Ra and Pb were scavenged with BaCrO_4_ precipitate, formed by adding 0.1 mL of 1.5 M Na_2_CrO_4_ solution. After centrifugation, the supernatant containing Sr was separated. To the supernatant, 1 mL of concentrated NH_4_OH and 2 mL of saturated (NH_4_)_2_CO_3_ solution were added to precipitate SrCO_3_. Lastly, the precipitate was dissolved in 10 mL of 1 M HNO_3_ (Solution B).

To distinguish the growth curve of ^90^Y from ^90^Sr during β-ray measurement, ^90^Y was removed by co-precipitation with Fe (OH)_3_. Fe^3+^ (2 mg) was added to 10 mL of Solution B. Concentrated NH_4_OH was then added until Solution B’s pH was 8–9. This step precipitated Fe(OH)_3_, which was used to scavenge ^90^Y. The supernatant was filtered from the precipitate using a glass microfiber filter (Whatman GF/F 25 mm, GE Healthcare Life Science Co.). Finally, Sr in the supernatant was precipitated as SrCO_3_ by adding 3 mL of saturated (NH_4_)_2_CO_3_ solution. The precipitate was filtrated with a membrane filter (JAWP02500, 25 mm diameter, Merck Millipore Co.), and stored in a stainless-steel sample dish (E0802001, 25 mm, 6 mm height, Chiyoda Technology Co.). The dish was covered with polyimide film (7.5 μm thick) to avoid further contamination and submitted for β-ray measurement.

Chemical yields of Sr, or the recovery of Sr carrier added to the sample solution at the beginning of the separation procedure, were 70% on average, with a range of 50–96% for 114 determinations.

### β-ray measurement

The β-rays emitted from ^90^Sr and its daughter ^90^Y were measured with a low background gas flow counter (LBC-4201B, Hitachi-Aloka Medical, Ltd.) for 3–12 h.

Following chemical separation of Sr, we monitored the growth of ^90^Y from ^90^Sr in SrCO_3_ precipitate. We measured β-rays 5 to 6 times within a fortnight of the separation. Using these measurements of radioactivity, we created a time-course plot to check whether the increase in β-ray counts fits the theoretical growth curve of ^90^Y. ^90^Y growth typically reached secular equilibrium with ^90^Sr 2 weeks after Sr separation, upon which we measured total radioactivity from ^90^Y and ^90^Sr. To correct for the self-absorption of β-rays by the measured sample, we used an absorption coefficient that was experimentally obtained with β-rays from known amounts of ^90^Sr and ^90^Y against the thickness of SrCO_3_ precipitate.

The background level of the gas-flow counter used in this study was 0.155 ± 0.015 cpm (3σ: 0.044 cpm) per 12 hr. Measurement efficiency was 0.339 ± 0.001 when the ^90^Sr standard sample was 88.4 Bq with a thickness of 24 mg cm^−2^. Assuming that the recovery of chemical separation of Sr is 70%, the detection limit of ^90^Sr in 1 g of incinerated sample is 4.94 mBq (g Ca)^−1^. All values obtained in this study were above this detection limit.

^90^Sr activity due to decay was corrected to March 11, 2011 (the day of the FNPP accident).

### Determination of stable Ca and Sr in the teeth

We determined Ca and Sr content in teeth using the inductively coupled plasma atomic emission spectrometer (ICP-AES; ICPE-9000, Shimadzu Co., Ltd.) at the Research and Analytical Center for Giant Molecules, Graduate School of Science, Tohoku University. Incinerated teeth samples were dissolved in 60% HNO_3_, and a portion of the solution was diluted 10,000 times for the measurements. Sr was determined using a standard addition method with a wavelength of 407.771 nm, and Ca was determined using a calibration-curve correction method with a wavelength of 317.933 nm. Each measurement was performed in triplicate and the resultant values were averaged.[Fig f4]

### Statistical analysis

Because the data were non-parametric, we chose Kruskal-Wallis tests for our analyses, with significance set at p < 0.05. Tests were one-tailed. Data on DM1, DM2, and DM3 were grouped together as deciduous molars (N = 16); data on M1, M2 and M3 were grouped as molars (N = 24); and data on P1, P2, and P3 were grouped as premolars (N = 21). Independent variables used in the test were area (H, L, C) and type of tooth (deciduous molars, molars, premolars). Dependent variables were ^90^Sr activity concentrations, ^90^Sr specific activity, and stable Sr concentrations. We used Spearman’s rank-order correlation analysis to look for significant correlations between ^90^Sr radioactivity in the soil and in the teeth. All analyses were performed in STATISTICA (Ver. 06J, StatSoft Co., Ltd.).

## Additional Information

**How to cite this article**: Koarai, K. *et al*.^90^Sr in teeth of cattle abandoned in evacuation zone: Record of pollution from the Fukushima-Daiichi Nuclear Power Plant accident. *Sci. Rep.* **6**, 24077; doi: 10.1038/srep24077 (2016).

## Supplementary Material

Supplementary Information

## Figures and Tables

**Table 1:** Characteristics of cattle used in current study.

Cattle code	Sampling sites	Birth	Age (months)	
			FNPP accident	Sampling
H-young-1	H	Jun. 16, 2010	8	25
H-young-2	H	Jul. 8, 2010	8	24
L-young-1	L	Jul. 8, 2010	8	17
L-young-2	L	Jul. 8, 2010	8	16
L-adult-1	L	Apr. 19, 2009	22	30
L-adult-2	L	Nov. 27, 2006	51	60
control-1	C	Jan. 9, 2011	2	24
control-2	C	Jan. 28, 2011	1	24

Area: The cattle residence location. Date of Birth: All subject cattle were born before the FNPP accident and had identifying ear tags with individually unique 10-digit numbers indicating their date of birth. Age: The ages of the cattle when the FNPP accident occurred and when they were euthanized (“sampling”).

**Table 2:** Stable Sr concentrations in incinerated tooth sample.

Cattle	Stable Sr concentration/μg g^−1^								
	Deciduous molars			Molars			Premolars		
	DM1	DM2	DM3	M1	M2	M3	P1	P2	P3
H-young-1	113 ± 1.4	106 ± 0.6	94.2 ± 0.6	122 ± 0.7	136 ± 0.4	157 ± 0.3	169 ± 0.4	153 ± 0.4	189 ± 0.3
H-young-2	193 ± 0.3	194 ± 1.0	206 ± 0.7	206 ± 1.2	238 ± 0.4	248 ± 0.4	255 ± 0.2	239 ± 0.4	241 ± 0.1
L-young-1	141 ± 1.8	128 ± 0.3	114 ± 1.1	122 ± 0.4	125 ± 0.8	159 ± 0.5	184 ± 0.5	166 ± 0.4	173 ± 1.2
L-young-2	**	133 ± 0.3	139 ± 0.5	147 ± 0.5	158 ± 1.3	214 ± 0.6	**	165 ± 0.8	**
L-adult-1	*	*	181 ± 0.3	187 ± 4.1	205 ± 0.7	204 ± 0.2	211 ± 0.7	213 ± 1.0	197 ± 1.3
L-adult-2	*	*	*	190 ± 0.7	155 ± 0.4	126 ± 0.9	137 ± 1.9	134 ± 0.8	121 ± 1.1
control-1	**	*	157 ± 0.1	145 ± 0.8	166 ± 0.4	163 ± 0.5	163 ± 0.2	163 ± 0.4	158 ± 0.1
control-2	214 ± 0.3	212 ± 0.7	233 ± 1.2	209 ± 0.6	184 ± 0.5	201 ± 0.5	201 ± 0.4	190 ± 0.8	181 ± 0.6

DM1: First deciduous molar; DM2: Second deciduous molar; DM3: Third deciduous molar; M1: First molar; M2: Second molar; M3: Third molar; P1: First premolar; P2: Second premolar; P3: Third premolar. Each sample was measured in triplicate. Data are shown as means ± SD.

*The tooth had already fallen out by the time of sampling.

**Data could not be obtained due to unsuccessful chemical separation of Sr.
