# Supplementary material for: 90Sr in teeth of cattle abandoned in evacuation zone: Record of pollution from the Fukushima-Daiichi Nuclear Power Plant accident
Source: Sci Rep. 2016 Apr 5;6:24077. doi: 10.1038/srep24077 (PMC4820720; doi:10.1038/srep24077)
Supplement: Supplementary Information [file srep24077-s1.pdf]

Supplementary information

^{90}Sr in teeth of cattle abandoned in evacuation zone: Record of pollution from the Fukushima-Daiichi Nuclear Power Plant accident

Kazuma Koarai[1], Yasushi Kino[1], Atsushi Takahashi[2], Toshihiko Suzuki[3, 4], Yoshinaka Shimizu[3], Mirei Chiba[3], Ken Osaka[3, 4], Keiichi Sasaki[3], Tomokazu Fukuda[5], Emiko Isogai[5], Hideaki Yamashiro[6], Toshitaka Oka[1, 7], Tsutomu Sekine[1, 7], Manabu Fukumoto[8], Hisashi Shinoda [*, 3]

[1] Department of Chemistry, Tohoku University, Japan

[2] Tohoku University Hospital, Tohoku University, Japan

[3]Graduate School of Dentistry, Tohoku University, Japan

[4] International Research Institute of Disaster Science, Tohoku University, Japan

[5] Graduate School of Agricultural Science, Tohoku University, Japan

[6] Faculty of Agriculture, Niigata University, Japan

[7] Institute for Excellence in Higher Education, Tohoku University, Japan

[8] Institute of Development, Aging and Cancer, Tohoku University, Japan

[*] Corresponding author.

E-mail: shinoda-h@m.tohoku.ac.jp

Address: 4-1 Seiryo-machi, Aoba-ku, Sendai 980-8575, Japan

Tel. & Fax.: +81 22 717 8275

Supplementary Table S1. ^{90}Sr activity concentrations in teeth.

Cattle	^{90}Sr activity concentration (mBq (g Ca)$^{-1}$)								
	Deciduous molars			Molars			Premolars		
	DM1	DM2	DM3	M1	M2	M3	P1	P2	P3
H-young-1	174 ± 22	89.0 ± 6.2	60.5 ± 4.4	81.0 ± 2.7	152 ± 6	385 ± 10	644 ± 33	367 ± 12	421 ± 14
H-young-2	115 ± 8	128 ± 6	188 ± 12	158 ± 10	675 ± 41	381 ± 31	831 ± 22	412 ± 12	431 ± 25
L-young-1	59.7 ± 8.2	52.0 ± 5.3	37.5 ± 4.9	57.2 ± 4.4	96.3 ± 7.8	167 ± 7	177 ± 12	311 ± 13	146 ± 12
L-young-2	**	43.1 ± 3.1	38.9 ± 3.0	65.3 ± 3.5	151 ± 5	177 ± 6	**	162 ± 6	**
L-adult-1	*	*	85.6 ± 9.4	91.1 ± 7.4	45.8 ± 5.3	73.7 ± 6.1	77.3 ± 13.6	62.1 ± 8.7	73.0 ± 15.0
L-adult-2	*	*	*	33.4 ± 5.9	36.1 ± 5.9	22.2 ± 3.6	30.8 ± 5.9	39.1 ± 6.6	59.8 ± 8.5
control-1	**	*	34.7 ± 3.1	18.1 ± 1.1	10.3 ± 1.2	6.3 ± 0.8	**	11.6 ± 4.1	10.6 ± 1.7
control-2	19.4 ± 3.4	12.8 ± 2.2	15.3 ± 2.3	12.6 ± 1.0	14.7 ± 1.1	14.3 ± 1.1	10.3 ± 5.2	14.7 ± 1.8	10.7 ± 1.6

DM1: First deciduous molar; DM2: Second deciduous molar; DM3: Third deciduous molar; M1: First molar; M2: Second molar; M3: Third molar; P1: First premolar; P2: Second premolar; P3: Third premolar.

* The tooth had already fallen out by the time of sampling.

** Data could not be obtained due to unsuccessful chemical separation of Sr.

Supplementary Table S2. ^{90}Sr specific activity in teeth.

Cattle	^{90}Sr specific activity (Bq (g Sr)$^{-1}$)								
	Deciduous molars			Molars			Premolars		
	DM1	DM2	DM3	M1	M2	M3	P1	P2	P3
H-young-1	536 ± 69	277 ± 19	212 ± 15	228 ± 8	383 ± 15	872 ± 22	1352 ± 69	845 ± 27	778 ± 26
H-young-2	214 ± 15	217 ± 11	301 ± 19	268 ± 17	1012 ± 62	544 ± 44	1169 ± 31	608 ± 18	617 ± 36
L-young-1	150 ± 21	135 ± 14	109 ± 14	160 ± 12	263 ± 21	374 ± 17	345 ± 17	641 ± 27	296 ± 24
L-young-2	**	107 ± 8	90.8 ± 7.1	145 ± 3.5	319 ± 11	282 ± 10	**	343 ± 12	**
L-adult-1	*	*	156 ± 17	166 ± 14	77.5 ± 9.0	129 ± 11	125 ± 22	99.6 ± 14	130 ± 27
L-adult-2	*	*	*	59.5 ± 10.6	78.1 ± 12.9	60.5 ± 9.9	76.3 ± 14.7	99.4 ± 16.8	166 ± 24
control-1	**	*	77.8 ± 7.0	44.3 ± 2.6	20.0 ± 2.3	13.4 ± 1.7	**	24.0 ± 8.4	22.8 ± 3.6
control-2	30.7 ± 5.3	20.2 ± 3.4	21.7 ± 3.3	19.9 ± 1.6	26.8 ± 2.0	24.4 ± 1.8	17.6 ± 8.9	26.8 ± 3.2	20.4 ± 3.0

DM1: First deciduous molar; DM2: Second deciduous molar; DM3: Third deciduous molar; M1: First molar; M2: Second molar; M3: Third molar; P1: First premolar; P2: Second premolar; P3: Third premolar.

* The tooth had already fallen out by the time of sampling.

** Data could not be obtained due to unsuccessful chemical separation of Sr.
